# Abscess pulsatility: a sonographic sign of osteomyelitis

**DOI:** 10.1186/s13089-023-00339-0

**Published:** 2023-10-03

**Authors:** Hope Werenski, Kristy Ford, Dillon Casey, Casey Glass, Jacob Schoeneck

**Affiliations:** Department of Emergency Medicine, Atrium Health Wake Forest Baptist, Wake Forest University School of Medicine, Medical Center Boulevard, Winston-Salem, NC 27157 USA

**Keywords:** Case report, Ultrasound, Soft tissue infections, Osteomyelitis

## Abstract

**Introduction:**

Early diagnosis and aggressive treatment of acute osteomyelitis may improve prognosis and prevent further complications. Sonography is useful in the evaluation of osteomyelitis. It can demonstrate early signs of inflammation, such as soft tissue changes near the affected bone, periosteal thickening, periosteal elevation, and subperiosteal abscess.

**Case presentation:**

A 68-year-old female presented to the emergency department with 3 weeks of worsening left lower extremity pain. She was initially seen by urgent care for left shin erythema and swelling and treated for cellulitis with intramuscular ceftriaxone without improvement. On presentation, she was afebrile and hemodynamically stable with erythema, swelling, and tenderness of the left pretibial soft tissues. Her labs revealed leukocytosis and elevated inflammatory markers. Point-of-care ultrasound demonstrated a bidirectional flow of fluid through a disruption in the bone cortex visualized on greyscale imaging and confirmed with color and spectral Doppler. The patient was diagnosed with osteomyelitis and treated with antibiotics and incision and drainage by orthopedic surgery.

**Discussion:**

The unique sonographic finding of pulsatile flow of fluid within an abscess near bone has not been previously described in the literature. The presence of Doppler signal in any fluid other than blood is known as pseudoflow. The presence of pulsatility in this case, which could represent either blood or pseudoflow, drew the ultrasound operator's eye to the cortical defect and lead to the diagnosis of osteomyelitis.

**Conclusions:**

The sonographic finding of pulsatility in an abscess near bone should raise the concern for communication with the medullary cavity.

**Supplementary Information:**

The online version contains supplementary material available at 10.1186/s13089-023-00339-0.

## Background

Acute osteomyelitis is a new-onset infection with progressive inflammatory destruction of bone [1]. Acute osteomyelitis is subdivided into hematogenous and nonhematogenous osteomyelitis, often resulting from contiguous spread or direct inoculation from trauma or surgery. Patients typically present with pain followed by swelling and erythema, which are indistinguishable from other soft tissue infections on exam. Fever is an uncommon sign [2]. The overall incidence of osteomyelitis in the United States is estimated to be as high as 50,000 cases annually [1]. Although the mortality rate of osteomyelitis is low, morbidity can be significant, ranging from the localized spread of infection to sepsis [3]. Early detection, diagnosis, and treatment is essential in minimizing complications such as systemic infection, permanent bone damage, and loss of function [4].

Problematically, early diagnosis of osteomyelitis requires a high index of suspicion and obtaining appropriate imaging studies [1]. A variety of standard imaging modalities, including x-ray, computerized tomography (CT), and magnetic resonance imaging (MRI), are used to visualize osseous changes in the setting of osteomyelitis [5]. Plain radiography and CT are limited by an inability to demonstrate inflammatory bony destruction in early osteomyelitis. MRI provides superior visualization of early changes in soft tissues and medullary structures and depicts early inflammation before later osseous changes. Despite the utility of MRI in early diagnosis of osteomyelitis, its use may be limited by scanner availability, time, and cost [6]. Point-of-care ultrasonography is rapid, non-ionizing, readily available, and can help detect signs of inflammation in acute osteomyelitis [7]. This report reviews a case of osteomyelitis presenting with the unique sonographic finding of pulsatile flow within an abscess.

## Case presentation

**Fig. 1:**
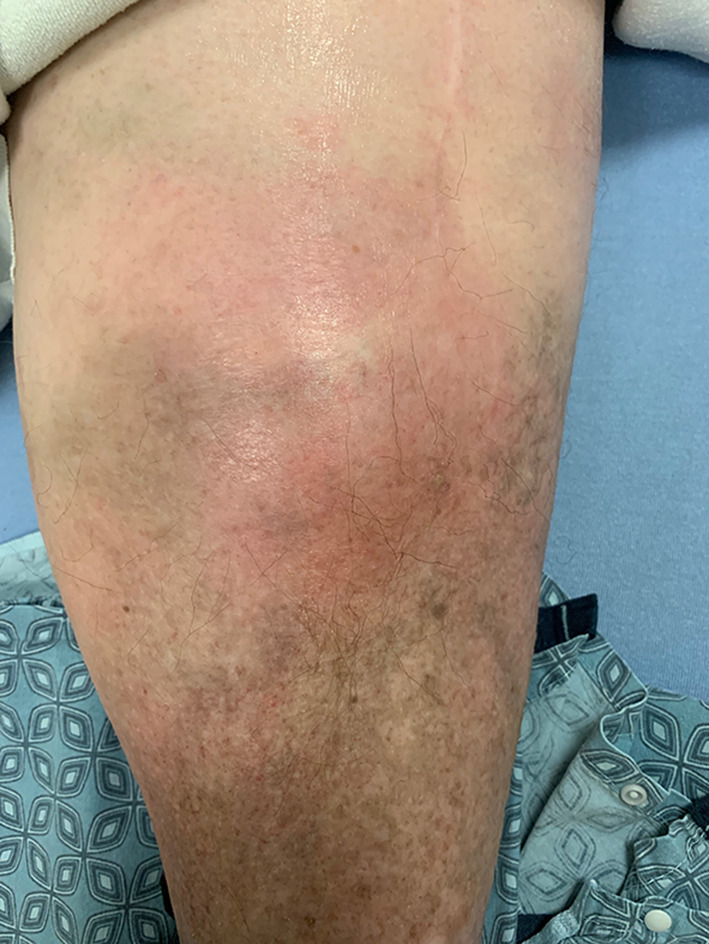
External appearance of the patient’s lower extremity demonstrating swelling and erythema

**Fig. 2:**
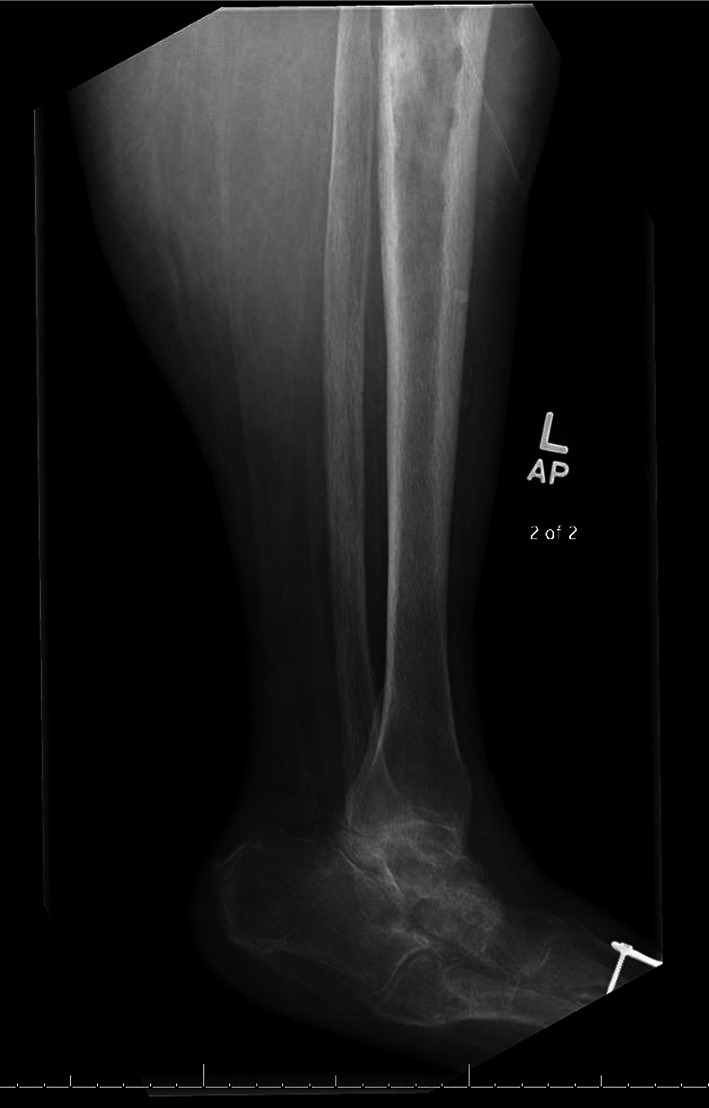
Plain radiograph of the patient’s left lower extremity demonstrating focal soft tissue swelling overlying the anterior aspect of the tibia

**Fig. 3:**
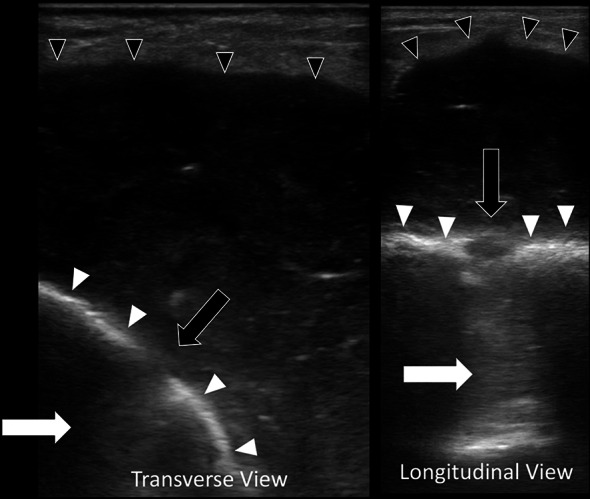
Point-of-care ultrasound demonstrating a heterogeneous hypoechoic fluid collection (black arrowheads) adjacent to the tibial cortex (white arrowheads). There is a defect in the cortex (black arrow) with fluid communicating into the medullary cavity (white arrow)

**Fig. 4:**
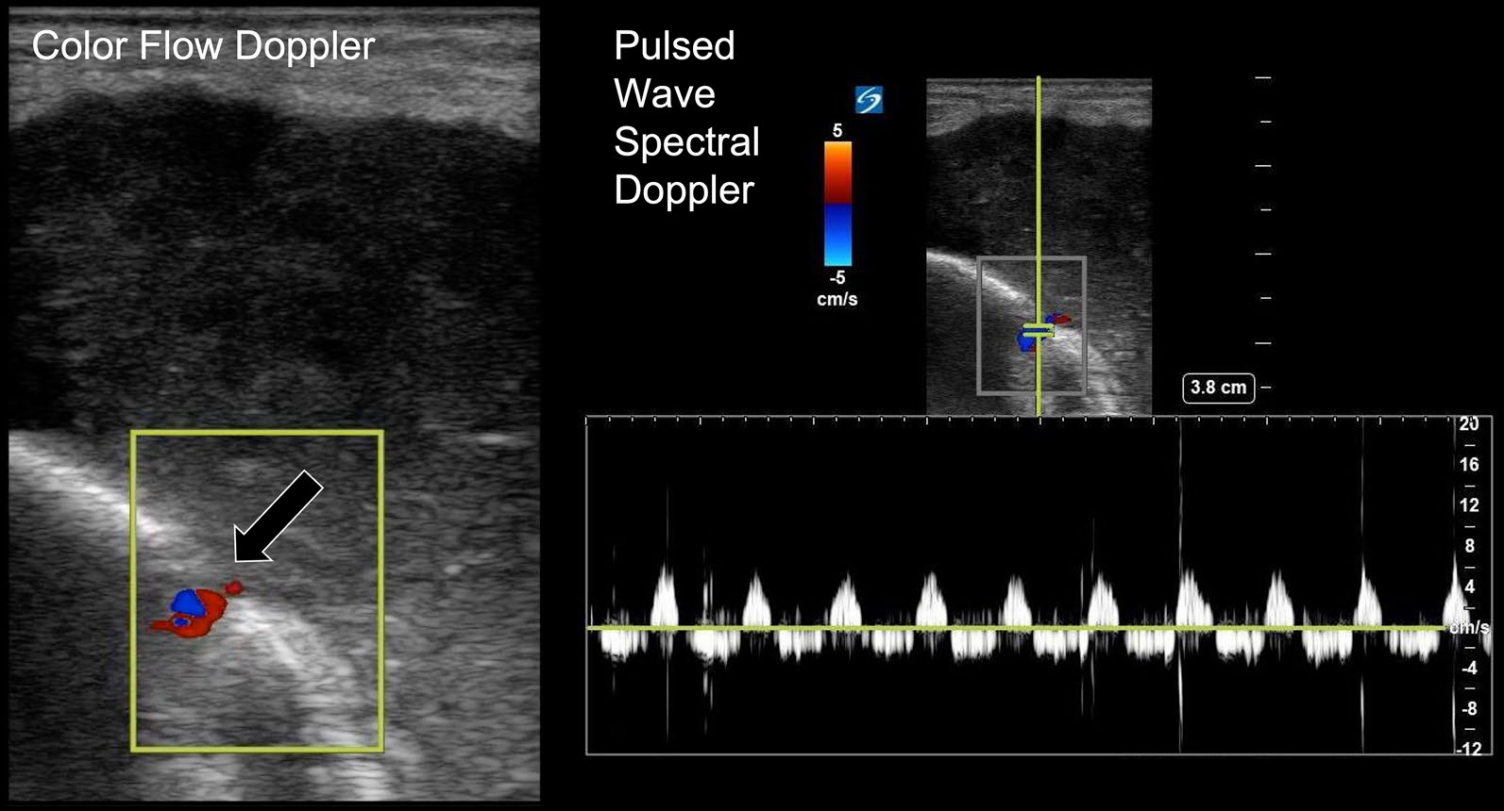
Point-of-care ultrasound demonstrating alternating color flow and pulsed wave spectral Doppler through the cortical defect (black arrow)

**Fig. 5:**
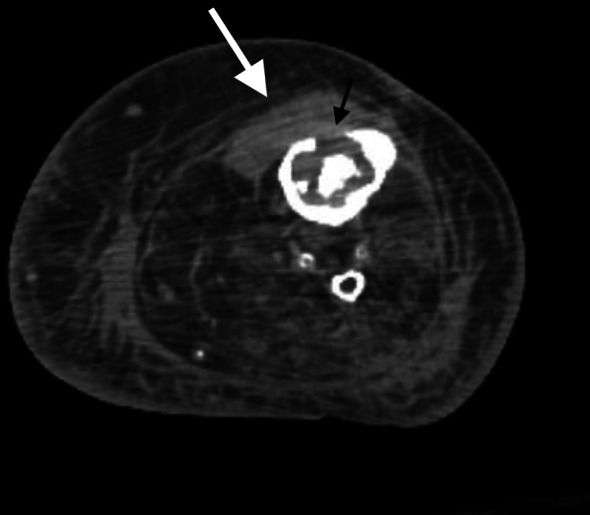
Computed Tomography of the patient’s left lower leg demonstrating a fluid collection adjacent to the tibia (white arrow) with erosion through the cortex (black arrow)

A 68-year-old female with a history of hypertension, hyperlipidemia, type 2 diabetes mellitus, gout, rheumatoid arthritis, and bilateral knee replacements on clindamycin prophylaxis for past infections presented to our emergency department with 3 weeks of worsening left lower extremity pain. She was initially seen at an urgent care for erythema and swelling of the left shin. Plain radiographs were unremarkable at that time and she was treated for cellulitis with intramuscular ceftriaxone for 10 days without improvement. On arrival, she was well-appearing, afebrile, and hemodynamically stable with erythema, swelling, and tenderness of the left pretibial soft tissues (Fig. 1). The area of erythema and swelling appeared to terminate before the knee joint and there was no appreciable joint effusion or limitation in range of motion on exam. Her pain was worse with ambulation, but she was able to bear weight on the affected leg. Repeat plain radiographs were performed and showed focal soft tissue swelling overlying the anterior aspect of the tibia (Fig. 2). A point-of-care ultrasound was performed by sliding the linear probe over the area of erythema in orthogonal transverse and longitudinal planes. Ultrasound demonstrated a large heterogeneous fluid collection adjacent to the tibial cortex (Fig. 3, Additional file 1: Video S1). There was a focal defect in the cortex and pulsatile fluid communicating with the medullary cavity. Alternating bidirectional flow was visualized with color flow and pulsed wave spectral Doppler (Fig. 4, Additional file 1: Video S1). The WBC count was normal (7.7), and systemic inflammatory markers were elevated (CRP 38 mg/dL, ESR 91 mm/hr). CT was obtained and demonstrated a 5.7 × 2.4 × 7.1 cm fluid collection adjacent to the tibial cortex with sinus tracts into the medullary cavity concerning for abscess with adjacent cellulitis and osteomyelitis (Fig. 5). She was given vancomycin and Piperacillin/Tazobactam, orthopedics was consulted, and she was admitted to the internal medicine service. An incision and drainage of the left pretibial abscess was performed. Neither wound nor blood cultures yielded any growth. Infectious Diseases was consulted and recommended treatment with vancomycin for 6 weeks.

## Discussion

Point-of-care ultrasound use was pivotal in this case, enabling the treating team to further characterize the extent of the patient’s soft tissue infection and reach the diagnosis of osteomyelitis. Bidirectional flow of fluid through a disruption in the bone cortex was visualized on greyscale imaging and confirmed with color and spectral Doppler. The pulsatile movement of fluid within an abscess has not been previously described in the literature. The presence of Doppler signal in any fluid other than blood is known as pseudoflow [8]. Additional examples include amniotic fluid, ascites, and ureteral jets [9]. The presence of pulsatility in this case, which could represent either blood flow or pseudoflow, drew the ultrasound operator’s eye to the cortical defect, raising the concern for bony involvement and the need for CT imaging and surgical consultation. Generalized hyperemia of infected tissues or direct compression of larger vessels within the medullary cavity could both be responsible for the presence of this pulsatility. The central arteries that supply the medullary cavity sustain a relatively high blood pressure to support flow to their many distant branches [10].

Previous studies have examined the utility of color and power Doppler to evaluate osteomyelitis, primarily in children [7, 11]. Doppler imaging typically reveals increased vascularization within or around the periosteum best visualized with power Doppler [12, 13]. Additionally, color and power Doppler have detected advanced osteomyelitis and demonstrated progression/regression of inflammation in response to antibiotic treatment [11]. Hyperemic hyper-vascular flow is usually described at the margins of periosteal abscesses, while internal flow without external compression would be considered atypical [13]. The internal flow observed in this abscess communicating with the medullary cavity may be unique to deep space infections that develop in confined compartments adjacent to vascular beds.

Several studies have demonstrated sonography’s ability to detect early inflammatory changes in acute osteomyelitis when correlated with clinical findings [6, 7, 11–15]. Soft tissue edema near the affected bone, tendon inflammation, synovitis, and joint effusion may indirectly indicate early osteomyelitis [12]. Sonographic changes suggesting osteomyelitis can be visualized within 1–3 days from the onset of symptoms [13]. Studies also suggest that sonography can demonstrate signs of inflammation from osteomyelitis earlier than plain radiography [6, 7]. These early sonographic findings progress to periosteal thickening, periosteal elevation with fluid adjacent to infected bone, and subperiosteal abscess. Cortical erosion is a late sonographic finding [7]. In the first 1–3 days of symptoms ultrasound is typically normal or shows mild soft tissue swelling adjacent to bone. Periosteal reactions develop within 4–7 days and later findings such as subperiosteal abscess or cortical erosion occur after 8–15 days of symptoms [16]. The previously derived test characteristics of ultrasound for osteomyelitis are variable, with sensitivities ranging from 55 to 76% and a specificity of 47% [5, 7, 16]. Ultrasound has been shown to have a higher sensitivity (74%) and specificity (95–100%) for complications of osteomyelitis, such as periosteal erosion or abscess formation [5, 7]. Ultrasound findings correlated with clinical findings and subsequent radiographic studies increase the accuracy of acute osteomyelitis diagnosis [5] and aid in monitoring the progression/resolution of disease [11].

Subperiosteal abscesses present as hypo- or anechoic lenticular-shaped fluid collections adjacent to bone cortex [9]. When performing point-of-care soft tissue ultrasound, the discovery of any fluid collections adjacent to bony surfaces should prompt further evaluation for osteomyelitis. Emergency medicine practitioners should avoid evacuating an abscess eroding through bone at the bedside and point-of-care ultrasound can help to avoid this pitfall. Fluid or tissue sampling from the affected region under sonographic guidance may eventually be needed to confirm the diagnosis of osteomyelitis and guide antibiotic treatment, but this should not be attempted without surgical consultation [6].

## Conclusions

Early detection of acute osteomyelitis facilitates the efficient collaboration of medical and surgical specialties, expediting debridement and antibiotic treatment. Sonography has several advantages in the evaluation of osteomyelitis, including rapid speed of performance, high portability, repeatability due to the absence of ionizing radiation, and the potential for earlier diagnosis. Several greyscale and Doppler findings associated with osteomyelitis have been previously described. The additional sonographic finding of pulsatile flow or pseudoflow in an abscess near bone should raise the concern for communication with the medullary cavity, prompting a search for cortical defects and obtaining more advanced imaging. The ability to detect bony involvement in an abscess on point-of-care ultrasound allows the clinician to proceed with appropriate management of osteomyelitis and avoid performing an incision and drainage at the bedside that should occur in the operating room.

### Supplementary Information

**Additional file 1. Video S1.** Photograph of the patient’s lower extremity, Transverse and longitudinal ultrasound clips demonstrating a heterogenous hypoechoic fluid collection with pulsatile flow through a cortical defect, Ultrasound clips demonstrating color and power Doppler flow, Spectral pulsed wave Doppler tracing with the sample volume placed to measure flow through the cortical defect, Computed tomography of the patient's extremity demonstrating an abscess.

## Data Availability

Data sharing is not applicable to this article as no datasets were generated or analyzed during the current study.
